# The need for information among patients with hematological malignancies: Psychometric analyses of the 62-item Hematology Information Needs Questionnaire (HINQ-62)

**DOI:** 10.1371/journal.pone.0201699

**Published:** 2018-08-09

**Authors:** Janneke A. J. Rood, Birgit I. Lissenberg-Witte, Corien Eeltink, Frank Stam, Florence J. van Zuuren, Sonja Zweegman, Irma M. Verdonck- de Leeuw

**Affiliations:** 1 Department of Hematology, VU University Medical Center, Amsterdam, the Netherlands; 2 Department of Internal medicine, Northwest Clinics, Alkmaar, the Netherlands; 3 Department of Epidemiology and Biostatistics, VU University Medical Center, Amsterdam, the Netherlands; 4 Department of Clinical Psychology, University of Amsterdam, Amsterdam, the Netherlands; 5 Department of Clinical Psychology, VU University Medical Center, Amsterdam, the Netherlands; IRCCS E. Medea, ITALY

## Abstract

The purpose of this study was to investigate the psychometric characteristics (content validity, internal consistency, and subscale structure) of the Hematology Information Needs Questionnaire-62 (HINQ-62), a patient reported outcome measure (PROM) for assessing the need for information among patients with hematological malignancies (HM-patients). Baseline data were used from a prospective study on the need for information which 336 newly diagnosed HM-patients had completed. In phase 1 (design phase), data from the first 135 patients were used and in phase 2 (validation phase), data from the remaining 201 HM patients were used. Content validity was analyzed by examining irrelevance of items. Items were considered irrelevant if more than 10% of the patients scored totally disagree on that item. The subscale structure of the HINQ-62 was investigated with Factor analysis (FA) (exploratory FA in phase 1 and confirmatory FA in phase 2). Cronbach’s α was computed for the different subscales and >.70 was considered as good internal consistency. None of the 62 HINQ-items were irrelevant. Exploratory FA identified five subscales: “Disease, symptoms, treatment and side-effects”, “Etiology, sleep and physical changes”, “Self-care”, “Medical tests and prognosis”, and “Psychosocial”. Root Mean Square Error of Approximation (RMSEA) among patients was 0.037 in phase 1 and 0.045 in phase 2. The comparative fit index (CFI)/Tucker-Lewis index -non-normed fit index among patients was 0.984/0.983 and 0.948/0.946, in phase 1 and 2 respectively. The internal consistency of the subscales was good, with Cronbach’s α 0.82–0.99. The HINQ is a valid PROM for assessing the need for information among Dutch HM-patients at diagnosis.

## Introduction

To optimize personalized medicine and care in patients with hematological malignancies (HM), it is essential to know these patients’ preferences regarding information provision. It is important to provide adequate information to cancer patients in general, as this has been found to be associated with better compliance with treatment[1] and better health related quality of life (HRQOL).[2–4] Moreover, adequate information to patients is a key element for shared treatment decision making.[5]

Detailed information on the perceived need for information among HM-patients is scarce[6], even though worldwide incidence of HM is growing. Annually, almost 920.000 patients are diagnosed with a HM[7] and the survival rate of most of these diseases has increased.[7,8] A recent literature review showed that information needs of HM-patients have only been assessed with study specific questionnaires and that no validated HM-specific information needs questionnaire is available. This hampers the comparison of information needs of HM patients across studies and the assessment of a particular patients’ information need in clinical practice.[6] On the basis of a cross-sectional study[9], we previously developed a first version a questionnaire, which comprised 92 questions regarding the need for information among HM patients.[9,10] Subsequently, using exploratory Factor Analysis (FA) and inter-item correlation, we shortened the questionnaire with 30 items, resulting in the HINQ-62. The aim of the current study was to assess the psychometric properties (i.e. internal consistency and validity) of the HINQ-62 among newly diagnosed patients with hematological malignancies.

## Patients and methods

### Study design

In this study we used the baseline data of a prospective study among newly diagnosed HM patients who had been recruited during two stages. During the first stage, from September 2013 to September 2014, patients were recruited at the in- and outpatient clinics of the VU University Medical Centre (VUmc), Amsterdam, Northwest Clinics, Alkmaar and Isala, Zwolle, the Netherlands. The second phase was conducted from September 2014 to July 2015 at the in- and outpatient clinics of the VUmc in Amsterdam, Northwest Clinics in Alkmaar, Isala in Zwolle, Westfriesgasthuis in Hoorn, and Spaarne Hospital in Hoofddorp, all in the Netherlands.

### Participants

Patients visiting the in- and outpatient clinics of the participating hospitals were asked to participate by a employee of the treating physician team if they were newly diagnosed (i.e. up to six weeks after diagnosis) with HM (acute or chronic leukemia, Hodgkin or Non-Hodgkin lymphoma, or multiple myeloma). Inclusion-criteria were: age ≥18 years and Dutch language proficiency. Exclusion-criteria were: concurrent treatment of another malignancy, terminal phase, and mental or physical inability to participate.

### Informed consent and procedure

This study was approved by the Medical Ethical Committee of the VU University Medical Center. Informed consent was obtained from all participants both orally and in written by a patient information file and informed consent form. Participants were asked to complete the questionnaires online or on paper. After two weeks, non-responders received a reminder, and after two more weeks non-responders were contacted by telephone by the first author or a study employee. This study was approved by the Medical Ethical Committee of the VUmc.

### The Hematological Information Need Questionnaire (HINQ)

In an earlier cross-sectional study[9], we developed the basic of this first version of the HINQ, which comprised 92 questions derived from three original validated questionnaires.[11–13] The Patient Learning Need Scale (PLNS), which measures the need for information of general medical or surgical patients about the topics that are especially important during discharge from hospital. The Toronto Information Needs Questionnaire-Breast Cancer, which measures the need for general information using various subscales and the Patient Information Needs Questionnaire, which assessed the information needs of cancer patients. These original and validated questionnaires were chosen because they 1) measure the need for information in various settings, and not only, for example, during palliative treatment, 2) have good validity and reliability, 3) require limited time for completion, and 4) are supplementary to each other. The third questionnaire (PINQ) was added, because it contains important items with an additional value, such as where to find good information on education material and literature, whether patients wanted to know their specific survival rates and their present condition, and whether patients want to know how to communicate with a physician. Items from these existing oncology questionnaires were supported with the ten categories of general cancer patients’ information needs in the literature[14]: cancer-specific, treatment related, prognosis, rehabilitation, surveillance and health, coping, interpersonal/social, financial/legal, medical system and body image/sexuality), and supported with a literature review on patients with hematological malignancies.[6] The English items were translated into Dutch by means of backward and forward translation by a native English speaker and the first and third author (native Dutch speakers) of this manuscript, and compared with the original English version. All items were discussed within an expert group of 8 people, consisting of internists, hematologists, clinical psychologists, and hematology nurse specialists, and the first author. The items were textually tailored to our HM patients. For example: TINQ-BC item 51 “When to have a mammogram” was changed into “When to have a bone marrow biopsy”.

In order to shorten the first version of the HINQ, we first performed an exploratory FA with the 92 items derived from data of our earlier cross-sectional study[9], which included 458 patients. FA with varimax rotation provided eleven factors with eigenvalues greater than 1, indicating an eleven-factor solution. Of these eleven factors, seven showed few loadings; therefore, we performed a forced factor analysis with four factors. Secondly, we computed inter-item correlations. With the expert group, we reached consensus to delete twenty-six items because they had a correlation of 0.75 or higher with two other items, and to delete another seven items due to overlap of content with other items. This overlap was confirmed by FA, in which these seven items loaded on the same factor. Slight disagreements were solved through discussion to generate one version. Furthermore, based on remarks of several patients, three items were added, one question each on need for information on fertility, on sexuality, and on concentration and memory problems.

The items of the resulting 62-item HINQ are scored on a Likert scale ranging from 1 (totally disagree) to 5 (totally agree) and a sixth option “not applicable”. Total scale scores were computed by summing up the item scores per scale and dividing the sums by the number of non-missing items, a higher score indicating a higher need for information.

### Psychometric analyses of the HINQ-62

The psychometric characteristics of the HINQ-62 were further investigated in two phases. Baseline data were used of a total of 336 newly diagnosed HM patients who participated in a prospective study on need for information. Data of the first 135 patients were used in phase 1 (design phase). In the validation phase, we used the data of the 201 HM patients recruited during the second stage (phase 2). Content validity was analyzed by examining irrelevancy of items. Items were considered irrelevant if >90% of the patients scored < 2 (i.e. totally disagree) on that item.

The factor structure of the HINQ-62 was analyzed with FA with varimax rotation of factors with eigenvalues >1.0. In phase 1, the structure of the HINQ-62 was investigated with exploratory FA. In phase 2, a confirmatory FA was used to analyze whether five-factor structure derived in phase 1 could be replicated. Criteria for an acceptable fit were root mean square error of approximation (RMSEA) <0.06, comparative fit index (CFI) and Tucker–Lewis index -non-normed fit index (TLI) ≥0.9.

The internal consistency of the subscales of the HINQ-62 was assessed with Cronbach’s alpha on the items belonging to that subscale, and values >.70 were considered good internal consistency.

## Results

### Study sample

In total, data was used from 336 newly diagnosed HM patients (135 in phase 1 and 201 in phase 2). The response rate of phase 1 was 69% and for phase 2 71%. The sociodemographic and clinical characteristics of the participants are presented in Table 1. The groups in phase 1 and phase 2 did not differ with respect to gender, age, educational level, nationality, membership of a patient association or hospitalization. Patients in phase 2 differed from those in phase 1 with respect to their diagnosis (p = 0.011), treatment intent (p<0.001), marital stage (p = 0.019) and type of hospital where the treatment was received (p<0.001) (See Table 1).

**Table 1:** Overview of sociodemographic and clinical characteristics of patients of phase 1 (n = 135) and phase 2 (n = 201).

		Phase 1		Phase 2		
		n	%	n	%	p-value
**Gender**	Female	53	39.3%	80	39.8%	0.92
	Male	82	60.7%	121	60.2%	
**Age**	Mean	58.8 (SD 15.6)		60.1 (SD 13.8)		0.64
**Marital status**	Unmarried	27	20.3%	22	10.9%	0.019
	Married	79	59.4%	148	73.6%	
	Cohabiting	9	6.8%	16	8.0%	
	Widowed	10	7.5%	9	4.5%	
	Divorced	8	6.0%	4	2.0%	
	Living with parents	0	0.0%	2	1.0%	
**Educational level**	Primary education	49	36.6%	75	37.7%	0.93
	Secondary education	46	34.3%	70	35.2%	
	Higher education	39	29.1%	54	27.1%	
**Nationality**	Dutch	132	97.8%	201	100.0%	0.064
	Otherwise	3	2.2%	0	0.0%	
**Diagnosis**	Acute Leukemia	28	20.7%	15	7.5%	0.011
	Chronic Lymphatic Leukemia	8	5.9%	20	10.0%	
	Chronic Myeloid Leukemia	13	9.6%	15	7.5%	
	Multiple Myeloma	24	17.8%	44	22.0%	
	Hodgkin Lymphoma	14	10.4%	20	10.0%	
	Non-Hodgkin Lymphoma	48	35.6%	86	43.0%	
**Treatment intent**	Curative	86	63.7%	77	38.5%	<0.001
	Non-curative	49	36.3%	123	61.5%	
**Comorbidity**	No comorbidity	73	54.1%	99	54.1%	1.0
	Mild comorbidity	37	27.4%	49	26.8%	
	Moderate comorbidity	20	14.8%	28	15.3%	
	Severe comorbidity	5	3.7%	7	3.8%	
**Treated hospital**	Academic	64	47.4%	51	25.4%	<0.001
	Non-academic	71	52.6%	150	74.6%	
**Hospitalized at time of the study**	No	124	93.2%	195	97.5%	0.057
	Yes	9	6.8%	5	2.5%	
**Member patient association**	No	126	94.7%	183	92.0%	0.33
	Yes	7	5.3%	16	8.0%	

### Content validity of the HINQ-62

On all HINQ-62 items, a need for information (score ≥ 2) was reported by more than 10% of the patients, indicating that none of the items were irrelevant or inapplicable. In 2.4% and 1.7% respectively item responses were missing (see Table 2).

**Table 2:** Mean (range) missing item responses of the HINQ-62 in phase 1 and 2.

	Phase 1		Phase 2	
	n	%	n	%
**Missing item responses**	2–4 (2.7)	1.5–3.1% (2.4%)	2–5 (3.2)	1.0–2.6% (1.7%)
**Response option: inapplicable**	10–57 (15.9)	6.9–39.3% (11.0%)	2–71 (12.1)	1.0–36.4% (6.2%)

### Subscale structure of the HINQ-62

Exploratory FA was used to investigate the subscale structure of the HINQ-62 in phase 1. We aimed to explain 70% of the variance with a restricted number of factors. Free FA provided nine factors with eigenvalues greater than 1.0. Since only a few items belonged to the factors six to nine, FA was forced into a five-factor solution. Item clustering on each factor was studied in relation to the factor structure that was proposed to emerge from the data to determine a conceptual interpretation of the factors. It appeared that the five factors could be labeled as 1) “Disease, symptoms, treatment and side-effects” (information regarding the disease, with its symptoms, the treatment and possible side-effects of treatment), 2)“Medical tests and prognosis” (information regarding the various tests, reasons and procedures of the tests, and prognosis of the disease and the influence of disease for the future), 3)“Self-care” (information regarding nutrition, social life and sport), 4) “Etiology, sleep and physical changes” (information regarding the illness and the etiology of the illness, regarding changes in physical appearance and sleep problems), and 5) “Psychosocial” (information regarding feelings, available help and communication with HCPs, family and others).

In phase 2, confirmatory FA showed an RMSEA of 0.037 (phase 1) and 0.045 (phase 2), which means a good fit. Furthermore, the CFI and TLI -non-normed fit index also showed a good fit (≥0.9) (see Table 3).

**Table 3:** Fit indices of the HINQ.

Phase	RMSEA^1^	CFI / TLI^2^
Phase 1	0.037	0.984 / 0.983
Phase 2	0.045	0.948 / 0.946

^1^ RMSEA<0.06 acceptable fit; <0.05 good fit

^2^ CFI/TLI≥0.9 acceptable fit; ≥0.95 good fit

### Internal consistency and factor loadings

The internal consistency of all HINQ-62 subscales in patients was high (>0.82) and is presented in Table 4. The factor loadings of all HINQ-62 items are presented in Table 5.

**Table 4:** Internal consistency (Cronbach’s α) of subscales of the HINQ.

Subscale	Phase 1	Phase 2
Disease, symptoms, treatment and side-effects	0.99	0.97
Medical tests and prognosis	0.97	0.91
Self-care	0.95	0.90
Etiology, sleep and physical changes	0.87	0.82
Psychosocial	0.90	0.90

**Table 5:** Factor loadings of the items on the Hematology Information Needs Questionnaire for study 1.

		Factor				
		1	2	3	4	5
1	What symptoms you may have related to your illness	0.70				
2	How the cancer acts in the body	0.81				
3	If there is cancer anywhere else in your body	0.79				
4	Your present condition	0.84				
5	The medical name for your type of cancer				0.55	
6	The cause of your illness				0.64	
7	If your illness is hereditary				0.57	
8	The possible course of your illness		0.74			
9	The reasons the doctor suggests certain tests		0.88			
10	How the tests are done		0.85			
11	Why they need to test your blood		0.79			
12	When to have a bone marrow biopsy		0.85			
13	What the results of your blood tests mean		0.86			
14	What types of treatment are available	0.63				
15	The treatment procedures	0.71				
16	How the treatment works against the cancer	0.70				
17	What the purposes of your treatment are	0.73				
18	How long you will be receiving treatment	0.67				
19	Why you need to take each medication	0.59				
20	When to take each medication	0.79				
21	The possible side effects of your treatment	0.60				
22	The possible reactions to each medication	0.54				
23	If there are ways to prevent treatment side effects	0.72				
24	What side effects you should report to the doctor/nurse	0.86				
25	If you are prone to infection because of your treatment	0.81				
26	What complications might occur from your illness	0.66				
27	Who to talk with if you hear about treatments other than surgery, radiation or chemotherapy				0.41	
28	How to manage the symptoms you may experience	0.86				
29	How to manage your pain	0.75				
30	If the treatment will alter the way that you look				0.63	
31	How much rest you should be getting				0.46	
32	How you can avoid stress				0.66	
33	What to do if you cannot sleep properly				0.51	
34	What to do if you have trouble urinating	0.70				
35	What to do if you have trouble with your bowels	0.74				
36	How to care for your wound or incision	0.69				
37	What you should do if you have problems with your memory or concentration	0.47				
38	Changes in the field of fertility				0.70	
39	Changes in the field of sexuality				0.69	
40	Possible results of your treatment	0.64				
41	How the illness may affect your life over the next few months		0.63			
42	How the illness may affect your life in the future		0.53			
43	If the cancer will come back		0.79			
44	Survival rates for your illness		0.76			
45	What you can do (or are allowed to do) in your situation (work, hobbies and social life)			0.76		
46	How to keep or become physically fit (exercises and diet)			0.61		
47	Which vitamins and supplements you should take			0.84		
48	Which foods you can or cannot eat.			0.79		
49	How to prepare the foods you are going to eat			0.67		
50	How to get through the “red tape” to get services at home			0.79		
51	Possibilities for your physical appearance during your treatment, e.g. wigs			0.45		
52	If there are groups where you can talk with other people with cancer					0.69
53	Who you should call if you have questions while you are still getting treatment	0.76				
54	What is the best way to talk or interact with a physician			0.57		
55	How to recognize your feelings toward your illness					0.72
56	Where you can get help to deal with your feelings about your illness					0.74
57	How to talk to family/ friends about your illness					0.69
58	How to tell if the cancer has come back					0.63
59	Opportunities for getting immediate help if you experience problems and have questions about your illness					0.57
60	What to do and who to talk to if you become concerned about dying					0.80
61	Where to get good educational material or literature about your illness or treatment	0.43				
62	Who you should call if you have questions after all the treatments are over	0.79				
	Eigenvalue	32.6	4.4	3.8	2.8	2.0
	Percentage variance explained	52.5	7.0	6.1	4.5	3.3

## Discussion

The aim of the current study was to assess the psychometric properties (i.e. internal consistency and validity) of the HINQ-62 among newly diagnosed patients with hematological malignancies. We developed the HINQ-62 to assess the need for information of HM patients in order to optimize individual patient information in clinical practice and to allow future comparison of data on information needs obtained from clinical trials. To our knowledge, we are the first to publish the development of a questionnaire (HINQ-62) assessing the need for information among patients with a hematological malignancy, and to investigate the psychometric properties of such a questionnaire. We validated the HINQ in a large population of patients who had different hematological malignancies, and were treated in academic as well as in non-academic centers, thus allowing a broad implementation in hemato-oncology care. The psychometric properties assessed were content validity, structure validity and internal consistency.

Analyses of the content validity of the HINQ-62 showed that none of the items of the HINQ-62 are irrelevant for assessing the need for information. The five factor structure of the HINQ-62 can be interpretated in a clinical meaningful way with the subscales: “Disease, symptoms, treatment and side-effects”, “Etiology, sleep and physical changes”, “Self-care”, “Medical tests and prognosis”, and “Psychosocial”. The internal consistency of the five subscales was high (Cronbach’s alpha > 0.82). Confirmatory FA derived from phase 1, also showed good fits in phase 2, thus validating the HINQ-62.

The HINQ-62, which has now qualified psychometrics and has been independently validated, can easily be implemented in daily clinical practice. We are of the opinion that this is important for several reasons. Firstly, data from the first subscale: “Disease, symptoms, treatment and side-effects” is important, because adequate information on the disease and its treatment has been found to lead to a better compliance with treatment.[15] In Chronic Myeloid Leukemia patients, non-adherence has been found to be predicted by a lower satisfaction with the information received.[15] Importantly, non-adherence was associated with a lower incidence of molecular remission of the disease and inferior clinical outcome.[16] In addition, information about the different treatment options is also important, because adequate information is the key for shared decision-making[17], as it is known to be associated with a better appraisal of the treatment decision-making by patients[5,18], greater satisfaction with treatment[19], better treatment adherence[20] and better HRQOL on various QOL outcomes.[21] Moreover, information on the cause of HM was found to be of added value for patients. It has been described that up to 59% of lymphoma and multiple myeloma survivors desired more information on the cause and course of the disease than they received.[22]

Secondly, it is important to address factors in the subscale, “Etiology, sleep and physical changes”, such as how to deal with fatigue. For instance, fatigue is a problem in many patients with HM[23]; it is associated with a decrease in HRQOL[24] and has negative effects on a patients daily life.[25]

Thirdly, information on “Self-care” including information on supportive care, is increasingly important in view of growing willingness and ability of cancer patients to manage on their own and to cope with the consequences of being treated for cancer by themselves.[26,27]

Fourthly, it is known that information about medical tests is one of the most often perceived needs among cancer patients[28], and prognostic information is rated as essential by HM patients[29–31]. However, this information should be tailored to the individual patient as a qualitative study by Friis showed that AML patients were given too much prognostic information that they did not ask for.[32]

The importance of the last subscale, “Psychosocial” confirmed by earlier studies that revealed that survivors of leukemia and lymphoma lacked information on support groups[30] and that survivors of lymphoma and multiple myeloma often experience a lack of psychosocial aftercare.[22] Psychosocial aftercare is particularly important among HM-patients, because they are among the patient groups with the highest psychological distress[33–35], for whom psychological information and support may be necessary during diagnosis, treatment and follow-up.

In clinical practice, the standardized and validated HINQ-62 may be used to ensure individualized information provision for HM patients. In addition, use of the HINQ-62 will ensure the comparability of the results of various studies on information needs for HM patients. By reviewing the literature[6] we have shown that this comparison is currently impossible, due to the use of different mostly self-made questionnaires and domains.[29,31,36–41] Furthermore, an important advantage of the HINQ-62 is that this shortened questionnaire will be faster to complete than the earlier version of the HINQ with 92 questions.[9,10] However, a 62-item questionnaire may still be too long for very sick patients and therefore further shortening of this PROM is warranted.

There are some limitations of this study. We included HM-patients at time of diagnosis only, which limits the generalizability to HM-patients during treatment, after treatment and during follow-up. Previous studies showed that HM patients may have different information needs during different phases of the disease.[14,42,43] Therefore, this questionnaire needs to be validated among HM patients in various phases of the disease, which we will do when the follow-up of the prospective study to the need for information from diagnosis to 18 months after diagnosis, is completed. A second limitation is that patients were not structurally involved in the initial development of the HINQ. However, during the second developmental phase of the HINQ-62, we asked patients to report any additional information needs, which were then implemented in the current version. A third limitation is that we did not perform a test re-test analysis. Future research might focus on shorterning the HINQ further for use in clinical practice and among HM-patients during treatment and follow-up.

## Conclusion

The HINQ-62 is a valid and reliable instrument for assessing the need for information among HM patients at time of diagnosis. This questionnaire will facilitate individualized information provision in clinical practice and will enable future comparisons between studies on the information needs of HM patients.

## Supporting information

S1 File Datafile to cohortgroup 1 and 2. (SAV)

S2 File Hematology Information Needs Questionnaire. (DOCX)
