# Supplementary material for: The need for information among patients with hematological malignancies: Psychometric analyses of the 62-item Hematology Information Needs Questionnaire (HINQ-62)
Source: PLoS One. 2018 Aug 9;13(8):e0201699. doi: 10.1371/journal.pone.0201699 (PMC6084926; doi:10.1371/journal.pone.0201699)
Supplement: S2 File — (DOCX) [file pone.0201699.s002.docx]

**Hematology Information Needs Questionnaire (English translation of Dutch version 1.0)**

1. What symptoms you may have related to your illness
2. How the cancer acts in the body
3. If there is cancer anywhere else in your body
4. Your present condition
5. The medical name for your type of cancer
6. The cause of your illness
7. If your illness is hereditary
8. The possible course of your illness
9. The reasons the doctor suggests certain tests
10. How the tests are done
11. Why they need to test your blood
12. When to have a bone marrow biopsy
13. What the results of your blood tests mean
14. What types of treatment are available
15. The treatment procedures
16. How the treatment works against the cancer
17. What the purposes of your treatment are
18. How long you will be receiving treatment
19. Why you need to take each medication
20. When to take each medication
21. The possible side effects of your treatment
22. The possible reactions to each medication
23. If there are ways to prevent treatment side effects
24. What side effects you should report to the doctor/nurse
25. If you are prone to infection because of your treatment
26. What complications might occur from your illness
27. Who to talk with if you hear about treatments other than surgery, radiation or chemotherapy
28. How to manage the symptoms you may experience
29. How to manage your pain
30. If the treatment will alter the way that you look
31. How much rest you should be getting
32. How you can avoid stress
33. What to do if you cannot sleep properly
34. What to do if you have trouble urinating
35. What to do if you have trouble with your bowels
36. How to care for your wound or incision
37. What you should do if you have problems with your memory or concentration
38. Changes in the field of fertility
39. Changes in the field of sexuality
40. Possible results of your treatment
41. How the illness may affect your life over the next few months
42. How the illness may affect your life in the future
43. If the cancer will come back
44. Survival rates for your illness
45. What you can do (or are allowed to do) in your situation (work, hobbies and social life)
46. How to keep or become physically fit (exercises and diet)
47. Which vitamins and supplements you should take
48. Which foods you can or cannot eat.
49. How to prepare the foods you are going to eat
50. How to get through the “red tape” to get services at home
51. Possibilities for your physical appearance during your treatment, e.g. wigs
52. If there are groups where you can talk with other people with cancer
53. Who you should call if you have questions while you are still getting treatment
54. What is the best way to talk or interact with a physician
55. How to recognize your feelings toward your illness
56. Where you can get help to deal with your feelings about your illness
57. How to talk to family/ friends about your illness
58. How to tell if the cancer has come back
59. Opportunities for getting immediate help if you experience problems and have questions about your illness
60. What to do and who to talk to if you become concerned about dying
61. Where to get good educational material or literature about your illness or treatment
62. Who you should call if you have questions after all the treatments are over
